# Food insecurity, environment, institutional quality, and health outcomes: evidence from South Asia

**DOI:** 10.1186/s12992-024-01022-2

**Published:** 2024-03-08

**Authors:** Mohammad Naim Azimi, Mohammad Mafizur Rahman

**Affiliations:** School of Business, University of Southern Queensland, Toowoomba, Australia

**Keywords:** Food insecurity, Health outcomes, Life expectancy, Mortality rates, Inflationary shocks

## Abstract

**Background:**

Food insecurity and environmental degradation pose significant threats to health outcomes in South Asia, necessitating effective policy interventions. Therefore, this study aims to examine the impact of food insecurity and environmental degradation on health outcome indicators amidst global inflationary shocks and institutional quality arrangements. Additionally, it aims to explore the intricate moderating role of institutional quality on the relationship between food insecurity, endogenous variables, and external shocks.

**Method:**

In alignment with the study’s objectives, a set of panel data spanning from 2000 to 2021 is compiled for South Asia. The study introduces a novel variable representing inflationary shock, crafted through the integration of inflation datapoints and the application of the generalized autoregressive conditional heteroskedasticity model. Additionally, a distinctive aggregate institutional quality index is formulated, drawing from six key measures of the Worldwide Governance indicators. To scrutinize the effects of food insecurity, environmental degradation, and other explanatory variables, the study employs the two-step system generalized method of moment technique, offering a robust analytical approach to uncover complex relationships and dynamics in the region.

**Results:**

The results indicate that the prevalence of undernourishment, inequality in per capita calorie intake, and CO_2_ emissions significantly reduce life expectancy and increase mortality rates. Additionally, it shows that per capita kilocalorie supply, per capita GDP, per capita health expenditures, and urbanization are statistically significant for increasing life expectancy and decreasing mortality rates. The findings reveal that inflationary shocks severely affect food insecurity and environmental factors, exerting further pressure on contemporary life expectancy and mortality rates. In rebuttal, the institutional quality index is found to have significant effects on increasing and decreasing life expectancy and mortality rates, respectively. Furthermore, the institutional quality index is effective in moderating the nexus between food insecurity, environmental degradation, and health outcomes while also neutralizing the negative impact of inflationary shocks on the subject.

**Conclusion:**

The results verify triple health constraints such as food insecurity, environmental factors, and economic vulnerability to global shocks, which impose severe effects on life expectancy and mortality rates. Furthermore, poor institutional quality is identified as a hindrance to health outcomes in South Asia. The findings suggest specific policy implications that are explicitly discussed.

## Introduction

**Fig. 1:**
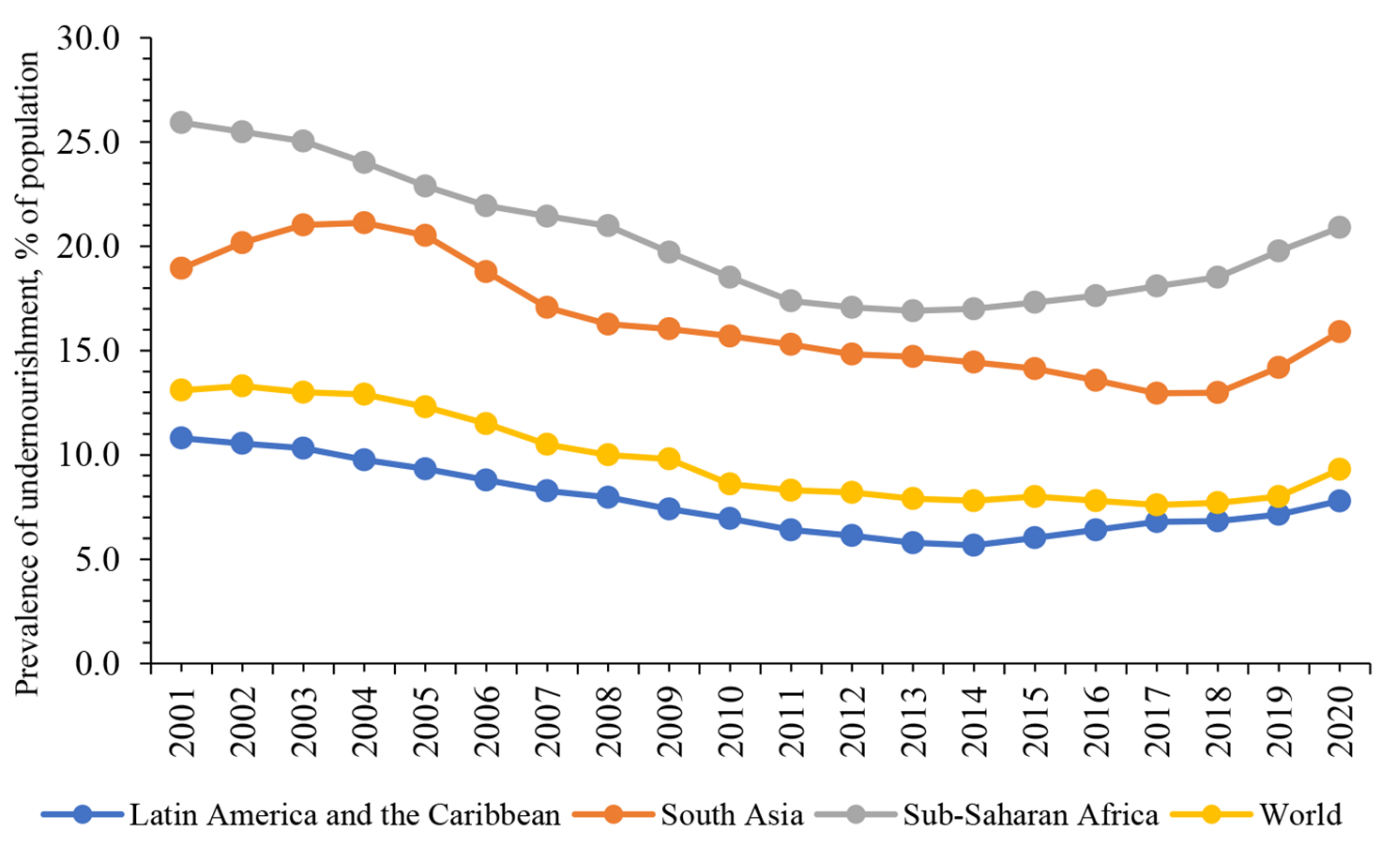
Prevalence of undernourishment (% of population) Source: Roser and Ritchie [5] in Our World in Data. The plot has been created by authors

Food insecurity (FI) remains an enduring global challenge, affecting over 9.3% of the world’s population. Nearly 29.6% of individuals currently face severe or moderate FI [1]. The World Bank [2] reports a substantial increase, with over 42% of the global population experiencing unaffordability of healthy food and basic nutritional needs in 2021, compared to 2019. FI is characterized by the inability to physically and economically afford and safe, nutritious food that meets dietary requirements for an active and healthy life [3]. Despite being one of the most fundamental human needs, having sufficient food to eat is a challenge in the real world. Figure 1 illustrates the prevalence of undernourishment, a key proxy for measuring food security (energy intake) worldwide. It highlights three highly undernourished regions—Sub-Saharan Africa, South Asia, Latin America and the Caribbean—compared to the global scenario. According to Fig. 1, Sub-Saharan Africa has the highest hunger rate at 20.9% in 2020, followed by South Asia (15.9%), and Latin America and the Caribbean (7.8%). Notably, South Asia, with its 15.9% hunger rate ranks second globally, exceeding the worldwide rate of 9.3%. As one of the most populous regions, South Asia is home to over 25% of the world’s population, a figure expected to rise by 40% in the next three decades [4].

**Fig. 2:**
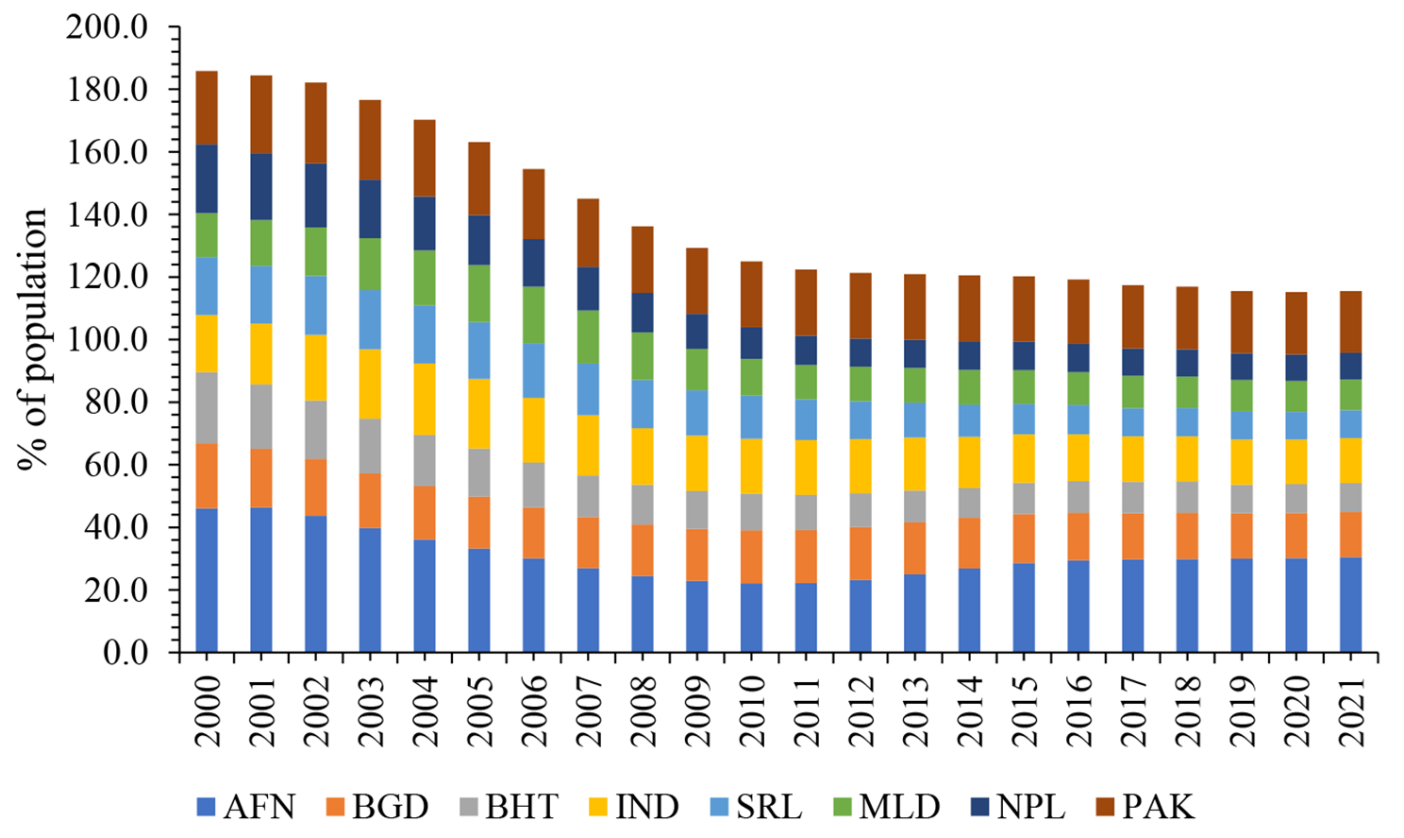
Prevalence of undernourishment; South Asia Notes: AFN: Afghanistan, BGD: Bangladesh, BHT: Bhutan, IND: India, SRL: Sri Lanka, MLD: Maldives, NPL: Nepal, PAK: Pakistan Source: Roser and Ritchie [5] in Our World in Data. The plot has been created by authors

At present, South Asia confronts a multitude of challenges, including draught, escalating food prices, environmental degradation, poverty, internal displacement of people, rapid population growth, high income inequality, and an alarming high prevalence of undernourishment [6]. Figure 2 illustrates that among South Asian countries, Afghanistan bears the highest rate of hunger, followed by Pakistan. Considerably, the rate of hunger has shown a narrowing trend in Nepal, the Maldives, and Bhutan in recent years. However, India and Bangladesh are anticipated to grapple with persistently high hunger rates in the years to come.

Furthermore, the region is home to over 40% of the world’s poorest inhabitants, with a headcount poverty ratio of less than $1.25 per day. While the global economy has been slowly recovering from the pandemics, recent political tensions have sparked a higher inflationary episode. Consequently, food prices have risen, and the supply of essential items such as wheat, barley, and sunflower oil has decreased. This surge in poverty stressors has limited access to food items, disproportionately affecting people globally and particularly in South Asia [7]. Evidence demonstrate the adverse effects of FI on human lives, including heightened exposure to chronic diseases, increased mortality rates, diminished mental stability, reduced human reproduction, and an elevated rate of miscarriage [8, 9]. Therefore, aside from managing the direct impact of FI on people’s well-being—with life expectancy and mortality rates being particularly noteworthy [10, 11]—governments and policymakers must address the broader challenge of mitigating the impact of global economic and inflation uncertainties on contemporary FI. This necessitates a comprehensive assessment of the influence of both endogenous FI indicators and the external shocks to pinpoint specific areas where precise policy tensions exist. Numerous studies [12–19] have explored the effects of FI on various health aspects, including life expectancy, mortality rates, chronic health diseases, and women’s pregnancy, across diverse geographical locations. While these studies and many others have predominantly focused on how endogenous predictors explain the subject, there has been a tendency to overlook the externalities that impose spillovers on health outcomes. For instance, Beyene [13] delved into the impact of FI on infant mortality rates and life expectancy in Sub-Saharan Africa. The study expanded on subject-endogenous predictors, including the prevalence of undernourishment, dietary energy supply, personal disposable income, and average schooling years. While these findings are noteworthy, their policy implications for precise policy reorientations may be limited. Therefore, the primary objective of our study is to explore the effects of FI and environmental degradation on health outcomes in South Asia, addressing a domain with an empirical dearth in the existing literature. While filling this gap is substantive for this investigation, the present study further aims to delve into specific areas of policy tension. Particularly, the study formulates five research questions of the present time: First, what is the impact of FI and environmental degradation on health outcomes in South Asia? Second, how do external (global) inflationary shocks impact health outcomes in the region? Third, does institutional quality impart meaningful direct and spillover effects on heath outcome indicators? Fourth, does institutional quality effectively moderate the relationships between FI and subject-endogenous variables? Fifth, does institutional quality modulate the negative effects of inflationary shocks on health outcome predictors in South Asia? Providing evidence-based answers to these questions is not only integral to achieving the study’s primary objectives but also crucial for identifying specific areas that necessitate targeted policy interventions.

The methodology and scope of the study make it a novel contribution to the existing literature. The distinctiveness of the present study’s contributions can be outlined as follows: Firstly, while a substantial body of literature has explored similar topics, South Asia has not been extensively examined in scholarly research, particularly, in the context of a precise and policy-oriented study. This study fills the gap by providing a focused exploration of the region. Secondly, a unique aspect of this study is the development of a novel inflation uncertainty predictor. This tool captures the impact of external inflationary episodes resulting from global political and trade tensions on health outcomes. This innovative strategy not only helps gauge the size and magnitude of the effects of sudden global price inflation but also provides guidance on policy interventions to absorb them. Thirdly, the study distinctively develops an institutional quality index using a distance-based technique. This index measures the influence of institutional quality on health outcomes, emphasizing the variability of exogenous forces that may affect contemporary health outcomes in South Asia. This approach is instrumental in understanding how existing governance responds to catastrophic food security and informs potential policy measures. Fourthly, in case where the existing governance structure does not directly respond to altering the subject, the study extends its analysis to investigate whether institutional quality plays a moderating role in improving the relationships between FI and health outcomes. This examination of the moderating role of institutional quality verifies the variability of macroeconomic, demographic, environmental, and FI predictors, thereby influencing health outcomes in South Asia. In sum, the conclusions drawn from the outcome of the study will enhance the current state of knowledge and help relevant policymakers in South Asian countries.

The subsequent sections of the study are structured as follows: Sect. 2 endeavors to conceptualize the paper and reviews pertinent empirical studies. Section 3 introduces the data, variables, and sources of data compilation. In Sect. 4, a foundational estimation method is established in alignment with the study’s objectives to test the competing hypotheses. Section 5 then presents the results and discusses the empirical findings. Finally, Sect. 6 concludes the article and offers specific policy implications.

## Literature review

### Conceptual framework

**Fig. 3:**
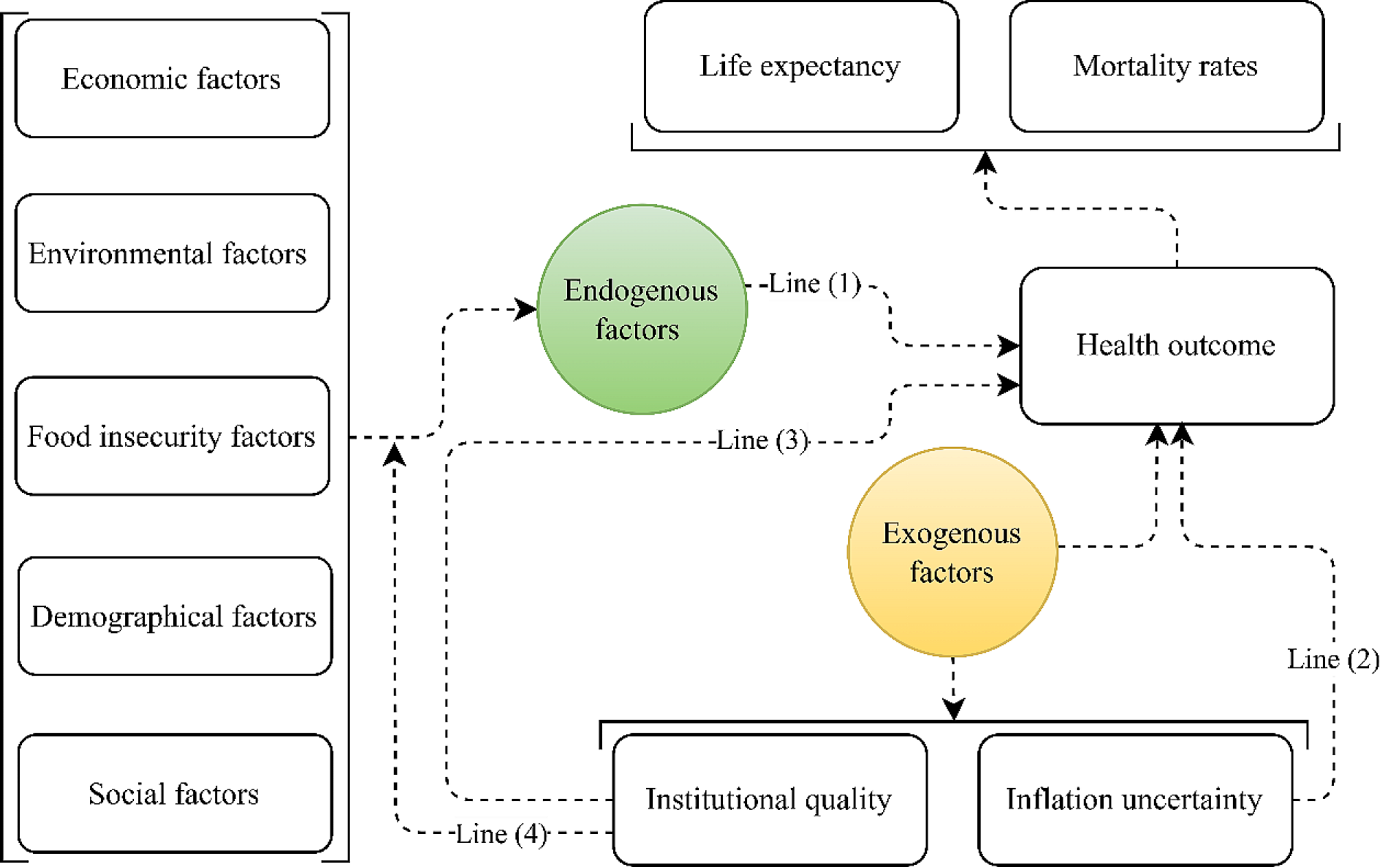
Study’s conceptual framework Source: Authors’ creation

Prior literature has predominantly focused on the health production function (HPF), establishing a conceptual framework that emphasizes endogenous factors such as health expenditures, per capita income, employment, environmental quality, lifestyle, education, and genetics [10, 20]– [22]. This approach traces back to the seminal work of Auster et al. [23], who explored the impact of environmental and healthcare indicators on mortality rates. However, despite the subsequent adoption of a similar pattern by substantial body of literature, most studies have overlooked exogenous factors. These factors include external social and economic shocks as well as institutions, which can either directly or indirectly influence health outcomes measured by mortality rate or life expectancy. In conceptualizing our study, we build upon this foundation, addressing the gap in literature. Figure 3 outlines the conceptual framework (extended HPF) designed for the present inquiry. Line (1) represents the conventional approach, as seen in studies like Onyimadu et al. [24] and Salgado et al. [25], offering for an extensive systematic review of studies examining how endogenous factors influence the subject. Line (2) illustrates how external shocks from global inflationary episodes, causing fluctuations in the general price level of food and non-food items, impact health outcomes. Line (3) outlines the direct effects of institutional quality on health outcomes, while Line (4) emphasizes the moderating effects of institutional quality on endogenous health factors.

### Institutional quality

Institutional quality represents the overall efficiency, reliability, and effectiveness of institutions in an economy [26]. Fundamentally, institutions encompasses rules, policies, and practices that form and instruct the behavior of individuals and organizations in a society [27–29]. Institutional quality is a multifaceted concept that gauges that state’s power to govern its resources for the benefit of the nation. According to the World Health Organization [30], states are responsible for designing a country’s health system based on two key pillars: resource production and efficient service provision. This design aims to facilitate the achievement of three objectives, including institutions’ responsiveness, health system efficacy, and the availability of sufficient and just financial and physical resources [31, 32]. To assess the efficacy and quality of institutions, Kaufmann and Kraay [33] developed six indicators, measuring corruption control, the rule of law, government effectiveness, political stability, regulatory quality, and voice and accountability. In essence, the higher these measures, the higher the institutional quality, signifying that a country has a robust and transparent mechanism in place to ensure the fair and efficient utilization of its resources, resulting to positive outcomes for the nations [34].

### Review of empirical studies

Our study aligns with prior empirical literature on several fronts, including the health consequences of economic growth, environmental degradation, demography, social factors, and institutional quality. For instance, researchers such as Dadgar and Norström [35], Gautam [36], Niu et al. [37], Spiteri and von Brockdorff [38], Erdoğan et al. [39], Salahuddin et al. [40], Knapp and Wang [41], and Mohapatra [42] have extensively examined the effects of economic growth, utilizing either per capita GDP or GDP growth rate, on health outcome indicators across diverse nations. They employed different statistical methods both at regional and country levels. The collective findings unanimously confirmed that economic growth plays a crucially positive role in influencing health outcomes. This positive influence operates through enhancement of individuals’ economic capacity, enabling them to afford better living conditions, accessing healthcare, and improve their living standards.

Furthermore, the review of literature reveals that numerous studies have delved into the relationship between environmental degradation and health outcome indicators, establishing a general concensus on the negative consequences of increased environmental degradation on population health. Noteworthy among these studies are Gasimli et al. [43], Mumtaz et al. [44], Omri et al. [45], Taghizadeh-Hesary et al. [46], Alimi et al. [47], Clark et al. [48], Li et al. [49], Emodi et al. [50], Zeeshan et al. [51], Murthy et al. [52], and Das and Debanth [53], which specifically explored the health consequences of CO_2_ emissions, ecological footprint, non-renewable energy consomption, and climate change predictors on mortality rates, life expectancy, and mental health of populations across various countries. Their collective findings consistently suggest that environmental degradation is detrimental to public health. Comparatively, FI, a sensitive topic of policy discussions worldwide, has not recived extensive scrutiny in the exisitng literature. The available studies, conducted by Beyene [13], Benzekri et al. [11], Johnson et al. [54], Dean et al. [55], Seligman et al. [56], Militao et al. [57], Pengpid and Peltzer [58], Nagata et al. [59], and Nwosu et al. [60] have explored the effects of FI on different health outcome indicators, including mental health, life expectancy, infant mortality rates, and per capita health expenditures. Using diverse data sources, these empirical investigations span different countries and regions, excluding South Asia. Despite this diversity, their unanemous findings support the assertion that FI poses an early-stage threat to human well-being, acting as a harbinger for various diseases over time.

While demography, often measured by population growth and urbanization, is considered a health-endogenous factor, recent emprical studies yield mixed responses. For instance, Jemiluyi [61], Tripathi and Maiti [62], Perrott and Holland [63], and de Meijer et al. [64] concluded that growing population rate and rapid urbanization have negative impacts on public health. Within a given per capita income, these factors increase contemporary health expenditures and suppress the overall health outcomes. Conversely, studies conducted by Huang et al. [65] and Shao et al. [66] argue that urbanization is an effective means of increasing life expectancy and reducting infant mortality rates by facilitating people with swift access to better healthcare facilities. Additionally, the review of existing empirical literature reveals that studies conducted by Liao et al. [67], Gumus and Yurumez [68], Raghupathi and Raghupathi [69], and Gottfried and Sublett [70] explored the health effects of social factors, primarily proxied by school enrollment rate across various countries, using diverse statistical methods for their analysis. In consensus, their findings emphasize that the level of education and literacy have a positive impact on life expectancy and negative effects on mortality rates. Finally, the study delved into existing literature and discovered that recent works conducted by Socoliuc et al. [71], Rahman and Alam [72], Vian [73], Van De Bovenkamp et al. [74], De Luca [75], Onofrei et al. [76], Glynn [77], Farag et al. [78], Rosenberg [79], Koller et al. [80], and Hadipour et al. [81], mostly employing the rule of law or control of corruption as proxies for institutional quality, affirm that institutional quality is crucially in promoting positive health outcomes. Essentially, they highlight the importance of governance structure and anti-corruption acts in contributing to the efficacy of healthcare systems.

### Research gaps

While recent empirical studies contribute significantly to existing literature, an added dimension would involve examining how contemporary health outcomes relate to externalities. Notably, there is a dearth of studies on the comprehensive effects of institutional quality on health outcomes, covering all aspects of the institutions both as a direct and moderating predictor. This gap is more tangible in the case of South Asian countries. Another gap is the absence, to our knowledge, of studies addressing global economic shocks, particularly global inflationary periods, which could significantly raise food prices, intensifying vulnerability to food security in South Asia. Moreover, apart from Gasimli et al. [43], who investigated the impact of environmental degradation on health outcomes, no other studies were found focusing on this aspect in South Asian countries. To address these gaps and align with our conceptual framework, we propose four key hypotheses: ***H***_***1***_: FI and environmental pollution have severe effects on health outcomes. ***H***_***2***_: Inflation uncertainty, as one of the key drivers of food price volatility, negatively impacts health outcomes. ***H***_***3***_: Institutional quality has a direct link with health outcome indicators. ***H***_***4***_: The interaction of institutional quality with inflation uncertainty increases or decreases the effects of endogenous health variables.

### Data and variables

Our study covers the period from 2000 to 2021, incorporating the latest available data. The empirical investigation centers on South Asian countries, including Afghanistan, Bnagladesh, Bhutan, India, the Maldives, Nepal, Sri Lanka, and Pakistan. The selection of South Asia as the context of our study is guided by two compelling reasons. Firstly, despite the abundance of studies on the health implications of FI and environmental degradation, the bloc has not received extensive attention in the existing literature. Secondly, the region is at a precarious equilibrium characterized by low staple productivity, minimal returns to formers, supply shortages, highly volatile food prices, area diversification, and low per capita income. These factors collectively contribute to escalating threats of FI on health outcome indicators, yet there is insufficient number of studies to guide contemporary policy directions for South Asia. Therefore, addressing these challenges necessitates a comprehensive analysis of the current situation to inform effective policies and resource reallocation in South Asia.

### Selection of variables

#### Dependent variables

The selection of the variables aligns with study’s objectives and is consistent with prior empirical literature [82–86]. We employ life expectancy at birth (*LE*), representing the number of years a newborn kid would survive if the prevailing mortality patterns at the time of its birth persist throughout its life. Additionally, we incorporate infant mortality rates (*MR*), indicating the number of kids who die before reaching one year of age per 1,000 live births per year. In this study, *LE* and *MR* are used as dependent variables. While *LE* represents a broader overview of a nation’s health outcomes, *MR* is considered as a micro-predictor. It is essential to examine the response of both macro- and micro-health outcome predictors to the explanatory variables.

#### Explanatory variables

In addition to two innovatively constructed variables for inflationary shocks and the institutional quality index (details in the next section), the study builds upon previous studies [13, 87]– [92] and employs three indicators, namely, prevalence of undernourishment (*PN*), per capita kilocalorie supply (*KS*), and inequality in per capita calorie intake (*CI*), as explanatory variables to measure FI in South Asia. *PN* is expressed as the percentage of people with insufficient regular food intake to maintain a typical, active life; a data value of 2.5 indicates a malnutrition rate lower than 2.5%. Moreover, *KS* represents the amount of all types of daily food supplies, measuring the available quantity of food for consumption. *CI* is expressed as the coefficient of variation of energy intakes, with a higher coefficient indicating greater inequality. These indicators are widely used in literature and are considered as best-fit proxies for measuring FI.

#### Control variables

To control for the effects of various social, economic, demographic, and environmental factors, the study gauges social factors through the school enrollment rate (*SE*), expressed as the gross percentage of enrollment in primary schooling to the total enrollment [83, 93]. *SE* is employed to capture the effects of education and literacy on the subject. Moreover, to account for macro-level economic variations and their effects on the dependent variables, per capita GDP growth (*PG*, annual %) is employed as a control variable [94, 95]. Per capita health expenditure (*HE*, constant 2015 US$) is utilized, following [96] and [97], to control for their effects on *LE* and *MR*. In this context, *HE* enables the assessment of the effects of out-of-pocket spending on the subject. Additionally, per capita CO_2_ emissions (CO_2_e), resulting from the use of fossil fuels and industry, serve as an environmental degradation variable [53]. Lastly, the study incorporates urbanization (*UR*, % of population) as a control variable for its effects on *LE* and *MR* [98, 99].

#### Construction of new variables

This part addresses the construction of the inflationary shock variable and institutional quality index. The persistent growth in the general price level of food items, especially when it is unpredictable in the future, cannot be overruled, Considering the previous period of inflationary episodes in South Asia that hindered general food prices, we innovatively construct a predictor of inflation uncertainty (*InF*). This allows for a more precise evaluation of the effects of economic variability on both *LE* and *MR*. In doing so, we use the datapoints of the annual inflation rate and the generalized autoregressive conditional heteroskedasticity (GARCH) model of Bollerslev [100] as follows:

1 $$VAR\left( {{\varepsilon _{INF,t}}} \right)=\sigma _{t}^{2}+{\vartheta _0}+{\vartheta _1}\varepsilon _{{INF,t - 1}}^{2}+\zeta \sigma _{{t - 1}}^{2}$$

**Fig. 4:**
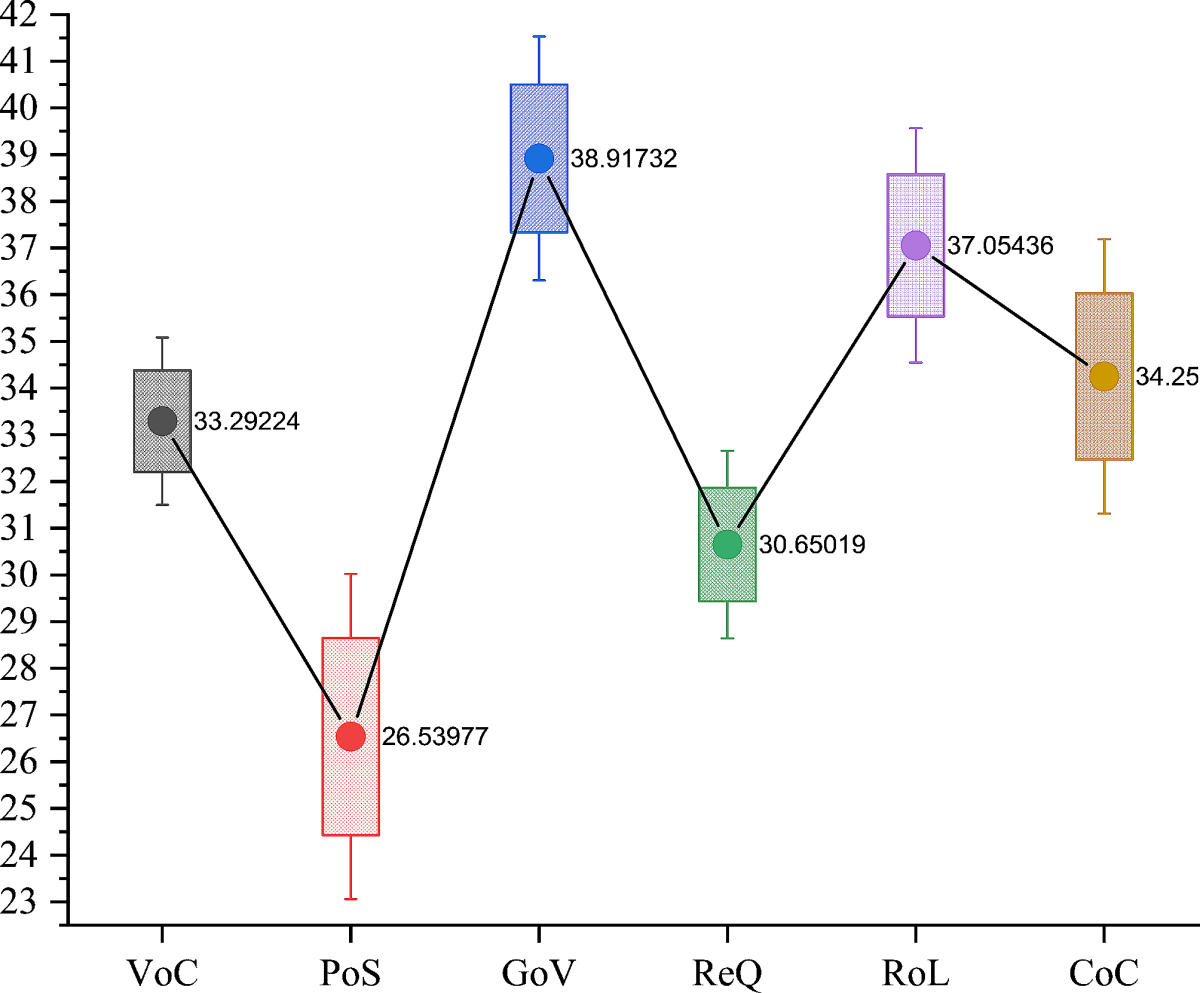
Institutional quality indicators Notes: VoC: Voice and accountability, PoS: Political stability, GeF: Government effectiveness, ReQ: Regulatory quality, RoL: Rule of law, CoC: Control of corruption. Data sourced from Worldwide Governance Indicators. Values are presented in percentile ranks from 1 to 100 (perfect)

In Eq. (1), $VAR\left({\epsilon }_{INF,t}\right)$ is the conditional variance of error term of the annual inflation rate, ${\vartheta }_{0}$ and ${\vartheta }_{1}$ are the intercept and autoregressive conditional heteroskedasticity parameter, respectively, and $\zeta {\sigma }_{t-1}^{2}$ represents the GARCH parameter. Additionally, since the 1980s, political instability, ineffective government, and, most importantly, corruption have been serious issues in South Asian economies that have brought local and international concerns to the fore [101]. This has been an alarming concern to most of the financial aid to uplift poverty, basic healthcare services, and FI. However, South Asian governments adopted programs of anti-corruption, they only remained as populist mottos. Based on the Worldwide Governance Indicators (see Fig. 4), though all institutional indicators are comparatively lower than other regions, political stability stands at 26.53 percentile rank, followed by regulatory quality at 30.65 percentile rank, voice and accountability at 33.29 percentile rank, rule of law at 37 percentile rank, and government effectiveness at 38.91 percentile rank. Values below the 50-percentile rank are alarming signs of poor institutional quality.

**Fig. 5:**
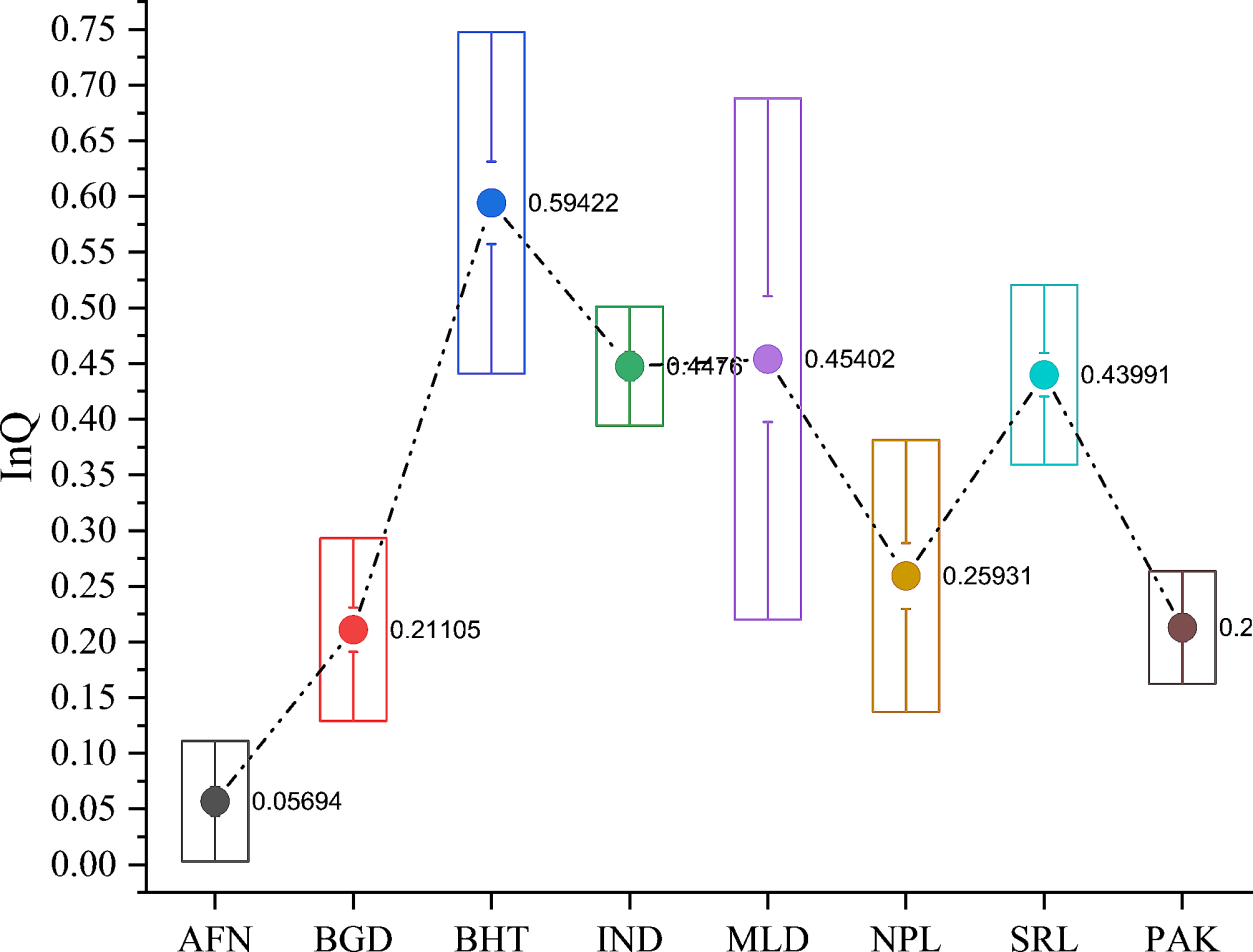
Cross-country institutional quality index (*InQ*). Notes: AFN: Afghanistan, BGD: Bangladesh, BHT: Bhutan, IND: India, MLD: Maldives, NPL: Nepal, SRL: Sri Lanka, PAK: Pakistan. InQ is expressed as numbers ranging from 0 to 1 (perfect) Sources of data compilation

Thus, to account for both the direct and moderating effects of institutional quality on the subject, we innovatively construct a comprehensive institutional quality index (*InQ*) following the distance-based approach proposed by Sarma [102]. This technique has recently gained prominence in the literature and has several advantages over common methods [103–106]. Figure 5 displays the constructed institutional quality index (*InQ*).

Initially, the study compiled relevant data at the country level and subsequently constructed a comprehensive panel for South Asia, encompassing 8 countries. The datasets for *LE*, *MR*, *SE*, *PG*, *HE*, *UR*, and annual CPI-based inflation rate were sourced from the World Development Indicators [107]. Additionally, the datasets for *PN*, *KS*, and *CI* were obtained from UN-FAO [108]. The data for per capital *CO*_2_e was sourced from the Global Carbon Budget, available in [109]. Finally, datasets for constructing the *InQ* have been compiled from Worldwide Governance Indicators [110] sources.

#### Estimation methods

Our primary objectives are to investigate how both endogenous and exogenous predictors influence health outcomes in South Asia. To that end, we modify the existing health production function using the lines of direction shown in our conceptual framework. First, to test the effects of FI, environmental factors, and other economic and social indicators in the presence of *InQ* and *InF* on health outcomes, we specify the following multivariate long-run equation:

2 $$\begin{gathered} H{O_{it}}=\delta +{\eta _1}P{N_{it}}+{\eta _2}K{S_{it}}+{\eta _3}C{I_{it}}+{\eta _4}S{E_{it}}+{\eta _5}P{G_{it}}+{\eta _6}H{E_{it}} \hfill \\ \,\,\,\,\,\,\,\,\,\,\,\,\,\,\,\,\,\,\,\,\,\,+{\eta _7}C{O_2}{e_{it}}+{\eta _8}U{R_{it}}+{\eta _9}In{Q_{it}}+{\eta _{10}}In{F_{it}}+{\wp _t}+{\varepsilon _{it}} \hfill \\ \end{gathered} $$

where all variables are defined before, *HO* refers to health outcome proxied by *LE* and *MR*, $$ \delta $$ is the intercept, and $$ {\eta }_{1}$$ to $$ {\eta }_{10}$$ are the long-run coefficients. Subscripts $$ i$$ represents the countries and $$ t$$ denotes time dimension. Equation (1) and the subsequent regressions account for country-specific unobserved fixed effects represented by $$ \wp $$. Finally, $$ {\epsilon }_{it}$$ presents the error term. To examine the moderating effects of *InF* and *InQ* on the relationship between health outcomes and the endogenous variables, we specify the following equation:

3 $$H{O_{it}}=\delta +\sum\limits_{{j=1}}^{{10}} {{\eta _j}{X_{it}}+} \,\theta \left( {{Z_{it}} \times {X_{it}}} \right)+\,{\wp _t}+{\varepsilon _{it}}$$

where $$ {\eta }_{j}$$ refers to the long-run coefficients of the explanatory variables $$ {X}_{it}$$ and $$ \theta $$ represents the long-run coefficient of the interaction term of the $$ {Z}_{it}$$ (*InF* or *InQ*) with the explanatory variables, using separate regressions for each interaction models. In order to incorporate the interaction terms into Eq. (3), we follow the same methodology as proposed by Abaidoo and Agyapong [111] and Dada and Ajide [112]. In doing so, we differentiate the health outcome indicators (*LE* and *MR*) with respect the explanatory variables as follows:

4 $$\frac{{\partial H{O_{it}}}}{{\partial {X_{it}}}}={\eta _j}+\theta {Z_{it}}$$

where the sign of $$ \theta $$ is a priori-indeterminant due to the expected effects (positive or negative) of the explanatory variables on *HO*. For example, we expect $$ \theta $$ to be positive in the relationship between *LE* and *KS* and negative in reducing the effects of *CO*_2_e on *MR*. To estimates Eqs. 2 and 3, we first consider the fixed effects (FE) model, where $$ \wp $$ is considered as the country-specific effects. The estimation of FE model is based on the assumption that $$ {\epsilon }_{it}$$ is correlated with $$ {X}_{it}$$ and uncorrelated with $$ \wp $$ [113]. Nonetheless, random effects (RE) model is an alternative to FE technique. It assumes that $$ \wp $$ is a random variable and uncorrelated with $$ {X}_{it}$$. If this assumption holds, then RE estimators would be more reliable than FE model [114]. This hypothesis can be tested using Hausman’s [115] specification approach. Additionally, the instrumental variables approach (*IV*) is another empirical competitor, which considers that there might be some exogenous variables, such as *InQ*, *InF*, and *CO*_*2*_e, as in our case, correlated with $$ {\epsilon }_{it}$$. It offers a mechanism to still estimate accurate and consistent coefficients. The *IV* regression takes the following form:

5 $$\begin{gathered} {Y_{it}}=\mu +{\vartheta _1}X_{{it}}^{1}+{\vartheta _1}X_{{it}}^{2}+\varepsilon _{{it}}^{1} \hfill \\ X_{{it}}^{1}=\mu +{\theta _2}X_{{it}}^{1}+{\theta _3}X_{{it}}^{2}+{\theta _4}X_{{it}}^{3}+\varepsilon _{{it}}^{2} \hfill \\ \end{gathered} $$

where $$ {X}_{it}^{1}$$, $$ {X}_{it}^{2}$$ and $$ {X}_{it}^{3}$$ refer to the endogenous variables, exogenous variables, and instrumental variables, respectively, $$ \theta $$ refers to the vector of reduced from coefficients, and $$ {\epsilon }_{it}^{1}$$ and $$ {\epsilon }_{it}^{2}$$ present the normal multivariate variance-covariance matrix. If the homoscedastic assumption holds true, then *IV* regression would be a good substitution. Nevertheless, in the presence of autocorrelation, cross-sectional dependence, heteroskedasticity, and endogeneity issues, neither of the above-cited models would be reliable. Therefore, to account for these issues, we estimate the *IV*-generalized method of moment (IV-GMM) model of Blundell and Bond [116]. It provides unbiased and consistent coefficients and has gained statistical prominence in prior literature. The IV-GMM model is also suitable for small samples like ours (*t* = 176), whether balanced or unbalanced [117]. Furthermore, unlike pooled OLS, FE, and RE techniques, the GMM model does not require the sample to hold normality assumptions [118]. For brevity, the moment conditions (MMs) of the GMM model, which were conducive to its development, take the following form:

6 $$E\left[ {{Z_{it}}{\varepsilon _{it}}\left( \vartheta \right)} \right]=E\left[ {{Z_{it}}\left( {{Y_{it}} - \left( \vartheta \right)X^{\prime}_{{it}}} \right)} \right]=0$$

where $$ {X}_{i,t-1}$$, $$ {X}_{i,t-2},$$ and $$ {X}_{i,t-i}$$ are the instruments used. The model can be estimated using system- or difference-GMM. The system-GMM model simultaneously includes two MMs for differenced and level equations, and it is evidently more accurate than difference-GMM [119, 120]. The difference-GMM, however, removes the fixed effects by differencing the employed data [121]. Further, the system-GMM is estimated using one-step or two-step estimators. Based on conventional asymptotics theory, however, estimators would be asymptotically normal in both approaches, but the two-step system-GMM estimator yields a comparatively smaller variance [122]. Therefore, the present study adopts the two-step system-GMM model. For estimation of the system-GMM, we used STATA/BE-17, in which the “*xtabond2”* command comes with a built-in diagnostic check for the first- and second-order autocorrelation, testing the well-being of the instruments used in the model under the presence of the first- and absence of the second-order autocorrelation [123]. Nevertheless, this inquiry does not aim to discuss the preference and technicality of the GMM model; the above-cited studies can be found highly informative.

## Results and discussions

### Summary statistics

**Fig. 6:**
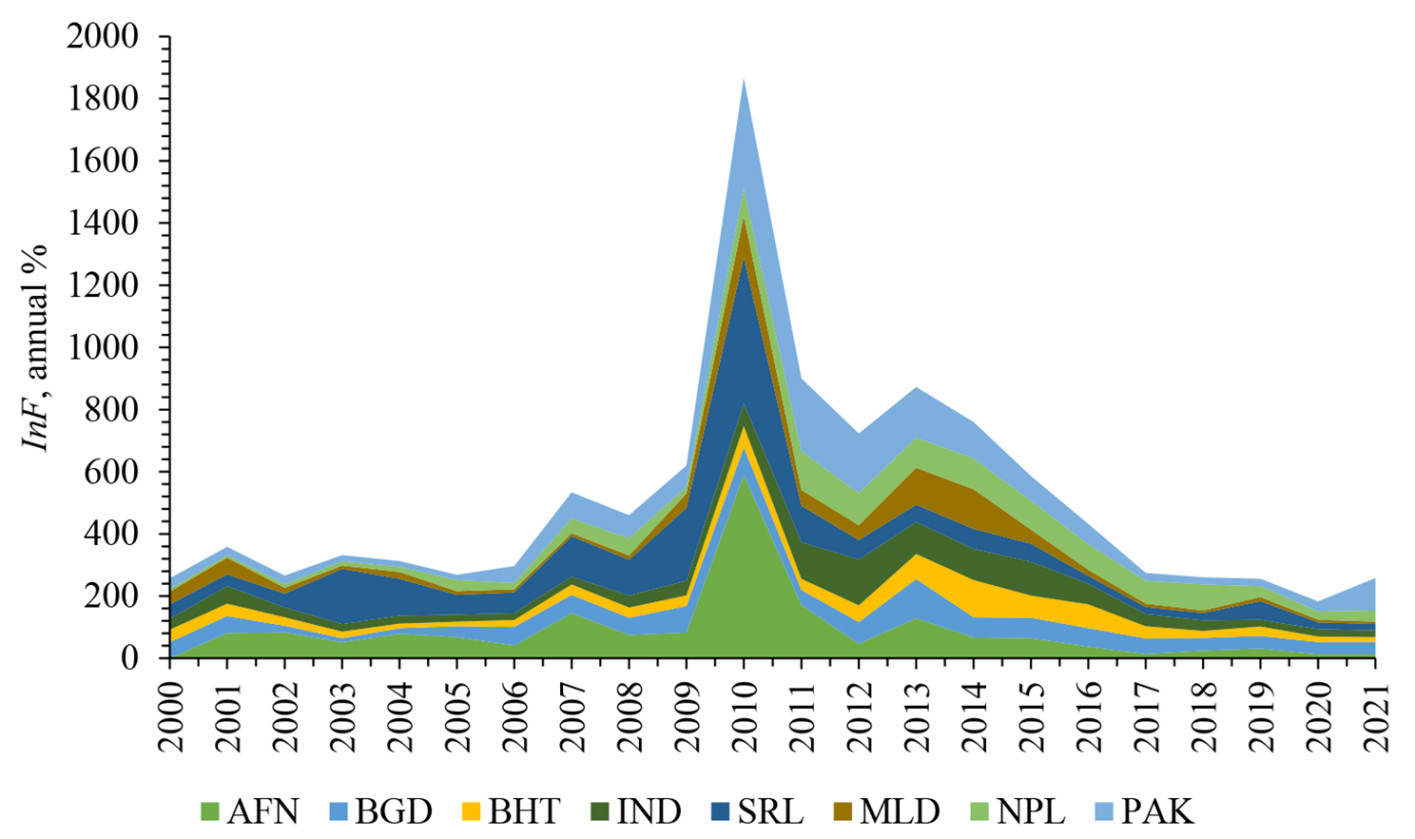
Cross-country *InF*, 2000–2021 Source: Authors’ depiction

This section presents summary statistics (see Table 1) for the variables utilized in the study. *LE* in South Asia averages 68.24 years, which is comparatively lower than East Asia (76 years) and Western Asia (72 years), yet similar to Central Asia (69 years). *MR* shows an average of 39.4 per 1,000 infants, with a maximum of 90.6 and a minimum of 22.3 in South Asia. This rate is higher than in other Asian regions; for instance, the average *MR* in East Asia has consistently remained between 12 and 13 per 1,000 infants throughout the recent decades. Worryingly, *PN* in South Asia is notably high at 17.37%, indicating a state of catastrophic FI. Afghanistan, Pakistan, Bangladesh, and India are at the top among the countries included in the study [5]. While for brevity, one may peruse other statistics, it is crucial to highlight the *InF* that averages 63.28% with a striking range from a minimum of 7.59% to a maximum of 589.7% over the years from 2000 to 2021. Again, Afghanistan, Sri Lanka, and Pakistan emerge as countries experiencing the highest levels of such uncertainties during 2010. Additionally, Fig. 6 illustrates the cross-country *InF* over the period under review. Finally, *InQ* reveals an average of 0.335, indicating a relatively low score across all institutional aspects. In practice, an average below 0.50 suggests catastrophic governance in an economy.

**Table 1:** Summary statistics

Variables	Obs.	Mean	Std. Dev.	Minimum	Maximum
LE	176	68.24	5.351	55.30	80.120
MR	171	39.408	22.342	5.100	90.600
PN	169	17.371	7.109	8.400	46.400
KS	176	17.212	2.372	12.55	21.060
CI	176	1.3021	1.023	1.271	1.447
SE	165	97.96	24.575	22.200	146.920
PG	176	2352.324	2594.008	280.33	10753.12
HE	170	119.009	204.436	8.340	993.47
CO_2_e	176	0.942	0.853	0.050	4.060
UR	176	28.447	7.818	13.400	43.010
InF	175	63.288	70.086	7.590	589.710
InQ	170	0.335	0.173	0.020	0.700

In addition, the study conducted a correlation analysis between the variables used, with the results presented in Table 2. The main purpose was to examine the potential presence of multicollinearity among the variables in the recipient panel. Two common approaches were applied for this purpose: Firstly, following Elith et al. [124], a threshold level of above 0.85 was suggested for detecting multicollinearity among the variables. Our results indicate that the correlation between all variables is below this threshold level. Secondly, the variance inflation factor (VIF) was calculated to further assess multicollinearity. The VIF, computed as a post-estimation of the pooled OLS model, reveals that all variables exhibit values less than 10, with a mean value of 4.10, below a threshold level of 5. Both methods unanimously confirm that the variables do not suffer from multicollinearity problems.

**Table 2:** Correlation matrix

Variables	(1)	(2)	(3)	(4)	(5)	(6)	(7)	(8)	(9)	(10)	(11)	(12)	VIF
(1) LE	1												
(2) MR		1											
(3) PN	-0.75	0.75	1										2.69
(4) KS	-0.48	0.47	0.28	1									4.85
(5) CI	-0.44	0.33	0.39	-0.03	1								1.96
(6) SE	0.55	-0.64	-0.58	-0.28	-0.16	1							1.94
(7) PG	0.62	-0.65	-0.42	-0.71	-0.32	0.38	1						6.10
(8) HE	0.71	-0.53	-0.32	-0.65	-0.21	0.32	0.72	1					7.25
(9) CO_2_	0.72	-0.51	-0.40	-0.44	-0.29	0.32	0.76	0.65	1				8.09
(10) UR	0.27	-0.00	-0.11	-0.09	-0.05	-0.07	0.36	0.38	0.62	1			2.51
(11) InF	-0.10	0.06	0.04	0.10	-0.26	0.00	-0.12	-0.11	-0.14	-0.11	1		1.23
(12) InQ	0.53	-0.54	-0.58	-0.36	-0.22	0.29	0.44	0.24	0.51	0.26	-0.20	1	4.38

### Insights into FI and LE nexus

Tables 3A and 3B report the results of 2Sys-GMM estimations. In Table 3A, column (1) reports the effects of FI on *LE*. Columns (2) to (9) further showcase the moderating effects of *InF* on FI and other explanatory variables. Table 3B, on the other hand, outlines the results of the moderating effects of *InQ* on the relationships between FI, *InF*, and other explanatory variables influencing *LE*.

The results reveal that a 1% increase in *PN* leads to a decrease in *LE* by 0.085 years in South Asia. This finding aligns with the observations of Beyene [13], who noted a decrease of 0.00348 years in *LE* with an increase in *PN* in Sub-Saharan Africa. Nutrition’s crucial role for the human body is emphasized, as sustained undernourishment can have significantly negative consequences on health [125]. Furthermore, the findings indicate that external shocks from *InF*, causing a surge in food prices, amplify the effects of *PN* on *LE*. The interaction of *InF* with *PN* reveals that a 1% increase in inflation forces *PN* to decrease *LE* by 0.091 years—an additional 0.006% point higher than the contemporary effects of *PN*. This resonates with the findings of Kidane and Woldemichael [126], who observed that a higher inflation rate diminishes people’s capacity to afford necessary food items, leading to long-term adverse consequences. Additionally, for every percent increase, *InQ* significantly contributes to increasing *LE* by 0.068 years. In Table 3B, column (1), the results underscore the highly effective moderating role of *InQ* in mitigating the negative impact of both *PN* and *InF* on *LE*. The interaction of *InQ* with *PN* demonstrates a reduction in the effects of *PN* on *LE* by 0.012 years. Consistent with our findings, Nugroho et al. [127] noticed that corrupted institutions serve as a concealed force contributing to the vulnerability of FI. They found that reducing corruption, meaning that people do not have to pay bribes, leads to improvements in undernourishment.

Additionally, the results highlight that *KS* significantly and positively contributes to an increase in *LE* by 1.749 years. However, the interaction of *InF* substantively diminishes the effects of *KS* on *LE* by 0.203 years. This underscores the highly negative impact of *InF* on the supply of necessary energy and food items in South Asia. Moreover, the interaction of *InQ* with *KS* reveals its effectiveness in enhancing the effects of *KS* on *LE* by 1.866 years. Importantly, it neutralizes the negative effects of *InF* (0.00054) on the subject. This suggests that while a higher inflation rate imposes elevated costs on agricultural inputs [128], leading to an overall increase in food prices, institutional quality may effectively mitigate or eliminate these inflationary stressors.

**Table 3A:** Effects of FI on LE

	(1)	(2)	(3)	(4)	(5)	(6)	(7)	(8)	(9)	
DV: LE	Effects of FI on LE	Effects of InF on PN-LE	Effects of InF on KS-LE	Effects of InF on CI-LE	Effects of InF on SE-LE	Effects of InF on PG-LE	Effects of InF on HE-LE	Effects of InF on CO_2_e-LE	Effects of InF on UR-LE	
Lagged DV	0.387*** (3.65)	0.407*** (4.15)	0.388*** (4.02)	0.343*** (3.87)	0.394*** (4.01)	0.391*** (3.99)	0.381*** (3.77)	0.369*** (4.18)	0.362*** (3.84)	
PN	-0.085** (-2.51)	-0.068* (-1.78)	-0.0889** (-2.30)	-0.094** (-1.94)	-0.086** (-2.44)	-0.085** (-2.36)	-0.087** (-2.43)	-0.091** (-2.58)	-0.090** (-2.51)	
KS	1.749*** (2.77)	1.535** (2.51)	1.63*** (2.60)	1.32*** (3.12)	1.56** (2.08)	1.708*** (2.73)	1.864*** (2.87)	1.847*** (2.86)	1.870*** (2.86)	
CI	-1.525** (-4.12)	-1.691* (-1.99)	-1.010** (-2.33)	-1.893* (-1.77)	-1.534* (-1.69)	-1.423** (2.19)	-1.098* (-2.23)	-1.127* (-1.64)	-1.488** (-2.10)	
SE	0.00964 (1.63)	0.00926 (1.46)	0.00959 (1.62)	0.00869 (1.47)	0.00151 (0.89)	0.00980 (1.33)	0.00902 (1.25)	0.00865 (1.26)	0.00979 (0.99)	
PG	0.0653*** (3.63)	0.0655*** (3.62)	0.0651** (2.60)	0.064*** (3.58)	0.063*** (3.54)	0.061*** (3.49)	0.067*** (3.53)	0.063*** (3.51)	0.064*** (3.53)	
HE	0.0257** (2.76)	0.0421* (1.69)	0.0232** (2.16)	0.056*** (4.04)	0.0263** (2.18)	0.0213* (1.72)	0.044*** (2.75)	0.023*** (3.16)	0.0133** (2.09)	
CO_2_e	-0.371** (-2.49)	-0.296* (-1.80)	-0.361* (-1.90)	-0.43*** (-4.10)	-0.27*** (-3.99)	-0.26*** (-4.31)	-0.29*** (-3.01)	-0.347* (-1.66)	-0.327** (-2.13)	
UR	0.168*** (2.96)	0.152*** (2.62)	0.166*** (2.92)	0.194*** (3.27)	0.150** (2.58)	0.154*** (2.98)	0.164*** (2.90)	0.168*** (2.95)	0.173*** (3.03)	
InF	-0.0632** (2.55)	-0.0385* (-1.67)	-0.0417** (-2.11)	-0.0341* (-1.87)	-0.05*** (-4.41)	-0.018** (-1.11)	-0.013*** (-2.9)	-0.0245* (-1.73)	-0.0419* (1.55)	
InQ	0.0688*** (4.11)	0.951*** (3.68)	0.0951*** (3.36)	0.0613*** (3.46)	0.0322*** (4.49)	0.0700*** (3.37)	0.0750* (1.70)	0.0827** (2.05)	0.0541*** (3.10)	
***Moderating effects***										
InF*PN		-0.091** (-3.99)								
InF*KS			0.203** (2.49)							
InF*CI				-2.031** (-3.36)						
InF*SE					0.00044 (0.94)					
InF*PG						0.0218** (2.37)				
InF*HE							0.0029*** (5.16)			
InF*CO_2_e								-0.464** (-2.61)		
InF*UR									0.236*** (5.51)	
Constant	-9.82*** (-8.02)	7.248*** (4.47)	7.867*** (5.13)	16.48*** (4.40)	7.175*** (3.66)	8.834*** (5.58)	11.18** (2.12)	10.03*** (3.99)	10.31*** (3.39)	
***Diagnostic checks***										
Observations	159	160	156	158	158	159	159	158	158	
Number of IDs	8	8	8	8	8	8	8	8	8	
Arellano-bond (1)	-3.67***	-3.91***	-4.01***	-3.62***	-3.85***	-3.90***	3.59***	-4.45***	-4.11***	
Arellano-bond (2)	-0.771	-0.902	-1.119	-0.682	-0.487	-1.008	-0.610	-0.804	-1.022	
Sargan chi^2^	29.13	33.08	33.12	33.01	30.45	31.87	30.46	33.15	31.21	

Notes: ***, **, and * indicate significance at 1%, 5%, and 10%, respectively. z-values are in parenthesis. DV: dependent variable, PN: Prevalence of undernourishment, KS: Per capita kilocalorie supply, CI: Inequality in per capita calorie intake, SE: School enrollment ratio, PG: Per capita GDP, HE: Per capita health expenditures, CO_2_e: CO_2_ emissions, UR: Urbanization, InF: Inflation uncertainty, InQ: Institutional quality index. InF*X refers to the interaction of inflation uncertainty with explanatory variables.

Source: Authors’ computations.

**Table 3B:** Effects of FI on LE

	(1)	(2)	(3)	(4)	(5)	(6)	(7)	(8)	(9)
DV: LE	InQ on PN-LE nexus	InQ on KS-LE nexus	InQ on CI-LE nexus	InQ on SE-LE nexus	InQ on PG-LE nexus	InQ on HE-LE nexus	InQ on CO_2_e-LE nexus	InQ on UR-LE nexus	InQ on InF-LE nexus
Lagged DV	0.247** (2.55)	0.382*** (3.97)	0.347*** (3.64)	0.380*** (3.95)	0.373** (2.58)	0.368*** (3.81)	0.379*** (3.95)	0.381*** (3.96)	0.390*** (4.04)
PN	-0.019*** (-3.45)	-0.081** (-2.26)	-0.116** (-2.88)	-0.082** (-2.32)	-0.079** (-2.11)	-0.084** (-2.38)	-0.0841** (-2.39)	-0.0881** (-2.46)	-0.0858** (-2.61)
KS	1.479*** (4.84)	1.211*** (3.18)	1.470** (2.63)	1.094** (2.18)	1.136** (2.56)	1.091*** (3.14)	1.249*** (3.01)	1.787** (2.34)	1.742*** (3.14)
CI	-1.95*** (-2.60)	-1.827* (-1.93)	-1.242* (-1.86)	-1.823* (-1.89)	-1.272* (-1.88)	-1.54*** (-3.11)	-1.115* (-1.99)	-1.283* (-1.89)	-1.358* (-1.90)
SE	0.0180 (1.08)	0.087 (1.14)	0.0654 (1.11)	0.076 (1.31)	0.0106 (0.94)	0.0119 (0.96)	0.0105 (0.87)	0.00961 (0.77)	0.0100 (0.88)
PG	0.0574*** (3.36)	0.071*** (3.85)	0.068*** (3.39)	0.067*** (3.41)	0.0399* (1.75)	0.0730*** (3.92)	0.0689*** (4.01)	0.0657*** (3.81)	0.0671*** (4.01)
HE	0.0388* (1.88)	0.084* (1.82)	0.0297* (1.92)	0.043** (2.11)	0.0101* (1.77)	0.0345* (1.99)	0.0603* (1.95)	0.0252* (1.99)	0.0275* (1.89)
CO_2_e	-0.694* (-1.91)	-0.489* (-1.90)	-0.350** (-2.10)	-0.39** (-2.42)	-0.561** (-2.10)	-0.615* (-1.91)	-0.079** (-2.46)	-0.356** (-2.50)	-0.332** (-2.47)
UR	0.158*** (2.99)	0.179*** (3.11)	0.191*** (4.00)	0.16*** (3.88)	0.184** (2.55)	0.177*** (3.18)	0.167*** (3.01)	0.170** (2.77)	0.155** (2.55)
InF	-0.0127** (-2.11)	-0.0689* (-1.79)	-0.058* (-1.80)	-0.0614* (-1.73)	-0.0713* (-1.77)	-0.076** (-2.22)	-0.0675* (-1.90)	-0.0633** (-2.46)	-0.0186** (-2.87)
InQ	0.083*** (3.60)	0.0309*** (3.22)	0.0201*** (4.45)	0.0621*** (3.65)	0.0642** (2.88)	0.0267*** (3.19)	0.0349*** (4.48)	0.088*** (4.00)	0.0871*** (3.71)
***Moderating effect***									
InQ*PN	-0.012*** (-3.04)								
InQ*KS		1.866*** (4.28)							
InQ*CI			-0.98*** (-3.11)						
InQ*SE				0.102 (1.01)					
InQ*PG					0.070*** (3.67)				
InQ*HE						0. 108*** (4.03)			
InQ*CO_2_e							-0.196** (-2.33)		
InQ*UR								0.490*** (3.43)	
InQ*InF									0.00054 (1.08)
Constant	-4.00*** (-3.89)	-7.48*** (-3.33)	-6.068** (-2.55)	-4.99** (-2.47)	-5.26** (2.45)	-4.52*** (-3.07)	-7.34*** (4.40)	-9.27*** (-3.18)	-9.55*** (-3.66)
***Diagnostic check***									
Observations	159	155	157	157	158	158	157	157	158
Number of IDs	8	8	8	8	8	8	8	8	8
Arellano-bond (1)	-4.55***	-4.13***	-4.33***	-4.66***	-4.65***	-4.02***	-4.88***	-4.78***	-4.10
Arellano-bond (2)	-0.999	-0.889	-1.812	-1.007	-1.045	-1.199	-1.099	-1.111	-0.861
Sargan chi^2^	28.12	31.45	30.55	32.11	30.61	32.21	29.20	30.44	28.16

Notes: ***, **, and * indicate significance at 1%, 5%, and 10%, respectively. z-values are in parenthesis. DV: dependent variable, PN: Prevalence of undernourishment, KS: Per capita kilocalorie supply, CI: Inequality in per capita calorie intake, SE: School enrollment ratio, PG: Per capita GDP, HE: Per capita health expenditures, CO_2_e: CO_2_ emissions, UR: Urbanization, InF: Inflation uncertainty, InQ: Institutional quality index. InQ*X refers to the interaction of institutional quality index with explanatory variables.

Source: Authors’ computations.

Consequently, this improves the relationship between *KS* and *LE*. These findings align with the work of Soko et al. [129], who similarly found that institutional quality has an effective mediating impact on the relationship between agricultural inputs and food security. The findings also reveal that *CI* has negative effects on *LE*. A 1% increase in *CI* reduces *LE* by 1.95 years. Again, the results highlight that when *InF* interacts with *CI*, the negative impact of *CI* intensifies, leading to a reduction in LE by 2.031 years. Conversely, the interaction of *InQ* with *CI* mitigates the negative impact of *CI* by 0.98 years. Notably, the findings do not support the significance of *SE* on *LE*. However, *PG* is identified as a positive factor affecting the dependent variable. The shock from *InF* reduces the positive effects of *PG* on *LE*, showing that *InF* reduces the purchase power of the people buying food items. In this context, *InQ* emerges as a significant moderating factor, countering the negative shock of *InF* on the nexus between *PG* and *LE*. These results support the findings of Salahodjaev and Chepel [130], Khan and Hanif [101], and Cicen [131], who observed that institutional quality modulates the negative impact of inflation rate on GDP. Furthermore, *HE* is found to improve *LE*. However, *InF* reduces the effectiveness of *HE*, while *InQ* improves the relationship between *HE* and life expectancy. Recent studies by Opeloyeru et al. [132] and Sharma et al. [133] also noticed that institutional quality improves the outcomes of health expenditures.

With respect to the environmental degradation effects, the results indicate that CO_2_e reduces *LE* in the recipient panel. Consistently, Azam et al. [82], Rahman et al. [134], and Majeed and Ozturk [135] support these findings on the negative impact of environmental degradation on *LE*. For instance, Azam et al. [82] delved into the effects of CO_2_e on *LE* in Pakistan and found that CO_2_e play a significantly negative role in reducing *LE*. Furthermore, the results show that the interaction of *InF* with CO_2_e increases its negative impact on *LE* from 0.371 to 0.484 years. The interaction of *InQ* with CO_2_e decreases its negative impact by 0.196 years. Ahmad et al. [136] investigated the effects of inflation instability on environmental degradation in Asian countries, and they similarly found that it hinders environmental quality and thus affects health outcomes. On the other hand, Jahanger et al. [137] provide support for the significance of institutional quality in improving environmental quality. Finally, the results offer statistical support for the positive effects of *UR* on *LE*. This association is grounded in the reality that individuals residing in remote areas often face constraints in accessing food, sanitation, and healthcare services, compared to their urban counterparts [98]. The challenges of poverty, unemployment, and illiteracy may further impede the quality of life for people in remote areas.

### Insights into FI and MR nexus

Tables 4A and 4B report the results of 2Sys-GMM estimations. In Table 4A, column (1) details the effects of FI on *MR*, while columns (2) to (9) report the moderating effects of *InF* on *MR* and other explanatory variables. Table 4B illustrates the moderating effects of *InQ* on the relationships between *MR*, *InF*, and other explanatory variables. The results reveal that *PN* significantly increases *MR*. Specifically, a 1% increase in *PN* leads to an increase in *MR* by 0.0845 per 1,000 infants. Contrastingly, *KS* is identified as a significant factor in reducing *MR*, with a decrease of 0.22 per 1,000 infants. However, *CI* is associated with an increase in *MR* by 0.74 per 1,000 infants. Notably, Banerjee et al. [85] studied the effects of FI on *MR* and found similar results. Moreover, Beyene [13] found that a 1% increase in *PN* statistically increases *MR* by 0.0119 per 1,000 infants in Sub-Saharan Africa. Our results show that compared to Sub-Saharan African countries, South Asia is more vulnerable to FI and experiences more deaths each year. Furthermore, the findings reveal that *PG* and *HE* are significant factors in reducing *MR*. It shows that a one US$ increase in *PG* causes *MR* to reduce by 0.012 per 1,000 infants. These results are consistent with those of Pérez-Moreno et al. [138], Salahuddin et al. [40], Kammerlander and Schulze [139], and Fotio et al. [140], who also found that economic growth is essential to reducing the contemporary *MR*. On the other hand, a one US$ increase in *HE* reduces *MR* by 0.260 per 1,000 infants. The results demonstrate that, compared to *PG*, *HE* is more effective in reducing *MR* in South Asia. Recent studies by Houeninvo [141], Schneider et al. [142], and Nketiah-Amponsah [143], have also observed that *HE* is significant in reducing *MR*. The interaction of *InF* shows that it highly reduces the impact of both *PG* and *HE* on *MR*, while *InQ* is found to effectively moderate the relationship between them. Similarly, Farag et al. [78] and Ahmad and Hasan [144] found that institutional quality plays an important moderating role in improving the nexus between *HE* and *MR*.

**Table 4A:** Effects of FI on MR

	(1)	(2)	(3)	(4)	(5)	(6)	(7)	(8)	(9)
DV: MR	Effects of FI on MR	Effects of InF on PN- MR	Effects of InF on KS- MR	Effects of InF on CI- MR	Effects of InF on SE-MR	Effects of InF on PG-MR	Effects of InF on HE-MR	Effects of InF on CO_2_e-MR	Effects of InF on UR-MR
Lagged DV	0.438*** (3.99)	0.434*** (4.01)	0.438*** (3.78)	0.440*** (3.88)	0.434*** (4.11)	0.437*** (3.65)	0.440*** (3.77)	0.436*** (3.96)	0.436*** (4.00)
PN	0.0845** (2.24)	0.0349* (1.86)	0.0912** (2.62)	0.0676* (1.85)	0.0109* (1.88)	0.0930* (1.90)	0.0141** (2.64)	0.0112* (1.99)	0.0114* (1.87)
KS	-0.220* (-1.91)	-3.300** (-2.37)	-3.178* (-1.99)	-3.048* (-1.88)	-3.220* (-1.94)	-3.261* (-1.89)	-3.005* (-1.87)	-3.311* (-1.93)	-3.303** (-2.10)
CI	0.740*** (4.09)	4.120*** (3.19)	3.714*** (3.07)	3.008*** (3.65)	3.479*** (3.26)	3.509*** (4.02)	3.141** (2.76)	3.076** (2.33)	3.388** (2.35)
SE	-0.036 (-0.42)	-0.0421 (-0.92)	-0.0363 (-0.88)	-0.0395 (-0.87)	-0.0856 (-0.99)	-0.0396 (-0.88)	-0.0355 (-0.91)	-0.047 (-1.15)	-0.0441 (-1.18)
PG	-0.012** (-2.11)	-0.0111* (-1.87)	-0.0125* (-1.94)	-0.0110* (-1.99)	-0.012** (-2.33)	-0.012** (-2.47)	-0.019** (-2.33)	-0.018** (-2.11)	-0.022** (-2.08)
HE	-0.260** (-2.65)	-0.256** (-2.39)	-0.260** (-2.11)	-0.252* (-1.92)	-0.243** (-2.71)	-0.258** (-2.14)	-0.255** (-2.14)	-0.257** (2.40)	-0.267** (-2.13)
CO_2_e	0.395*** (4.18)	0.374*** (3.15)	0.394* (1.92)	0.362** (2.44)	0.356*** (3.88)	0.412*** (4.12)	0.376*** (4.22)	0.419*** (3.02)	0.464*** (3.03)
UR	-0.302* (-1.99)	-0.318** (-2.14)	-0.293** (-2.13)	-0.226** (-2.37)	-0.267** (-2.45)	-0.315** (-2.09)	-0.244** (-2.31)	-0.316** (-2.17)	-0.380** (-2.18)
InF	0.0618** (2.18)	0.030*** (2.26)	0.0890*** (3.05)	0.0120*** (3.59)	0.0531*** (3.12)	0.0816** (2.28)	0.0864* (1.97)	0.0146** (2.20)	0.0546** (2.51)
InQ	-0.091** (-2.53)	-0.034** (-2.81)	-0.086** (-2.15)	-0.078** (-2.63)	-0.061** (-2.37)	-0.075** (-2.19)	-0.040** (-2.34)	-0.058** (-2.29)	-0.014** (-2.20)
***Moderating effects***									
InF*PN		0.00131* (1.84)							
InF*KS			-3.880** (-2.66)						
InF*CI				4.046*** (4.11)					
InF*SE					-0.00128 (-1.03)				
InF*PG						-0.009** (-2.18)			
InF*HE							-0.0005* (-1.91)		
InF*CO_2_e								0.441* (1.99)	
InF*UR									0.000180 (0.902)
Constant	5.810*** (3.01)	7.520*** (4.45)	5.012*** (3.65)	5.861*** (4.82)	6.315** (4.14)	6.583*** (3.65)	5.739*** (4.24)	7.604*** (3.11)	7.704*** (4.77)
***Diagnostic checks***									
Observations	159	160	156	158	158	159	159	158	158
Number of IDs	8	8	8	8	8	8	8	8	8
Arellano-bond (1)	-3.88***	-4.13***	-3.98***	-4.39***	-4.01***	-3.89***	-4.110***	-3.91***	-4.17***
Arellano-bond (2)	-0.904	-1.017	-0.844	-1.021	-0.902	-1.014	-0.888	-1.091	-0.855
Sargan chi^2^	27.33	30.44	30.37	27.91	30.01	29.89	31.01	28.42	31.76

Notes: ***, **, and * indicate significance at 1%, 5%, and 10%, respectively. z-values are in parenthesis. DV: dependent variable, PN: Prevalence of undernourishment, KS: Per capita kilocalorie supply, CI: Inequality in per capita calorie intake, SE: School enrollment ratio, PG: Per capita GDP growth, HE: Per capita health expenditures, CO_2_e: CO_2_ emissions, UR: Urbanization, InF: Inflation uncertainty, InQ: Institutional quality index. InF*X refers to the interaction of inflation uncertainty with explanatory variables.

Source: Authors’ computations.

**Table 4B:** Effects of FI on MR

	(1)	(2)	(3)	(4)	(5)	(6)	(7)	(8)	(9)
DV: MR	InQ on PN-MR nexus	InQ on KS-MR nexus	InQ on CI-MR nexus	InQ on SE-MR nexus	InQ on PG-MR nexus	InQ on HE-MR nexus	InQ on CO_2_e-MR nexus	InQ on UR-MR nexus	InQ on InF-MR nexus
Lagged DV	0.443*** (8.35)	0.443*** (5.45)	0.447*** (4.87)	0.439*** (4.55)	0.442*** (4.16)	0.446*** (7.01)	0.450*** (5.12)	0.447*** (4.76)	0.443*** (4.09)
PN	0.0128** (2.30)	0.058*** (3.88)	0.0123** (2.26)	0.0837* (1.83)	0.0872** (2.14)	0.0944* (1.92)	0.0118** (2.10)	0.0122* (1.78)	0.011** (2.28)
KS	-2.630* (-1.67)	-2.064** (-2.11)	-2.542** (-2.09)	-3.079* (-1.85)	-2.683** (-2.10)	-2.555* (-1.69)	-2.389** (-2.01)	-2.675** (-2.45)	-3.077* (-1.77)
CI	3.225*** (3.31)	0.219** (2.32)	0.364*** (3.05)	0.655*** (3.38)	0.446* (1.89)	0.480** (2.19)	0.503*** (3.26)	0.448*** (3.38)	0.427** (2.33)
SE	-0.0370 (-0.78)	-0.0304 (-1.19)	-0.0233 (-0.88)	-0.0531 (-0.69)	-0.041 (-1.10)	-0.0342 (-0.88)	-0.0201 (-0.66)	-0.0337 (-0.82)	-0.0395 (-1.09)
PG	-0.142** (-2.16)	-0.156* (-1.74)	-0.125** (-2.14)	-0.105* (-1.83)	-0.114** (-1.75)	-0.286** (-2.33)	-0.154** (-1.73)	-0.134** (-1.69)	-0.111** (-1.83)
HE	-0.248** (-2.10)	-0.207** (-2.16)	-0.229*** (-4.00)	-0.237* (-1.77)	-0.241* (-1.78)	-0.215* (-1.74)	-0.265** (-2.27)	-0.234** (-2.45)	-0.240* (-1.99)
CO_2_e	0.361* (1.77)	0.248*** (4.04)	0.375* (1.68)	0.416** (2.12)	0.325*** (3.50)	0.417** (2.67)	0.275*** (4.11)	0.780** (2.53)	0.543* (1.81)
UR	-0.210** (-2.28)	-0.257* (-1.65)	-0.223*** (-4.11)	-0.154* (-1.80)	-0.136** (-2.20)	-0.245* (-1.95)	-0.752** (-2.03)	-0.140** (-2.00)	-0.265** (-2.14)
InF	0.056*** (4.00)	0.522*** (3.59)	0.563* (1.90)	0.532** (2.36)	0.537*** (3.58)	0.543*** (4.01)	0.600** (3.89)	0.528* (1.70)	0.470*** (3.77)
InQ	-0.0771** (-2.23)	-0.0679* (-1.90)	-0.0205** (-2.17)	-0.0654* (-1.87)	-0.026** (-2.22)	-0.067** (-2.15)	-0.0503* (-1.92)	-0.065** (-2.11)	-0.0427* (-1.88)
***Moderating effects***									
InQ*PN	0.0044* (1.69)								
InQ*KS		0.424** (2.45)							
InQ*CI			1.693 (0.89)						
InQ*SE				-0.000022 (-1.10)					
InQ*PG					-0.362** (-2.44)				
InQ*HE						-0.143** (-2.18)			
InQ*CO_2_e							0.031 (1.36)		
InQ*UR								-0.124** (-2.11)	
InQ*InF									0.0067 (1.012)
Constant	5.191*** (9.33)	5.805*** (8.14)	13.322*** (6.15)	12.339*** (4.36)	6.011*** (3.98)	4.360*** (4.45)	4.087*** (8.10)	5.019*** (6.21)	5.369*** (3.17)
***Diagnostic checks***									
Observations	159	160	156	158	158	159	159	158	158
Number of IDs	8	8	8	8	8	8	8	8	8
Arellano-bond (1)	-4.33**	-4.28***	-4.35***	-4.19***	-3.97***	-4.08***	-4.25***	-4.21***	-4.01***
Arellano-bond (2)	-1.019	-0.852	-1.355	-1.099	-1.389	-1.206	-1.117	-0.906	-1.007
Sargan chi^2^	30.87	31.90	32.38	30.45	34.17	34.09	31.49	30.99	32.67

Notes: ***, **, and * indicate significance at 1%, 5%, and 10%, respectively. z-values are in parenthesis. DV: dependent variable, PN: Prevalence of undernourishment, KS: Per capita kilocalorie supply, CI: Inequality in per capita calorie intake, SE: School enrollment rate, PG: Per capita GDP growth, HE: Per capita health expenditures, CO_2_e: CO_2_ emissions, UR: Urbanization, InF: Inflation uncertainty, InQ: Institutional quality index. InQ*X refers to the interaction of institutional quality with explanatory variables.

Source: Authors’ computations.

Additionally, the results indicate that CO_2_e have positive impacts on *MR*, showing that an increase in environmental degradation increases *MR*. *InF* is found to increase the effects of CO_2_e, while *InQ* is observed to have a substantive moderating role in neutralizing the negative impact of *InF* and CO_2_e on *MR*. It is obvious in most of the South Asian countries. For example, due to financial incapacity, people burn plastic, tyers, and rubbers for heating purposes during winter. Our results are consistent with those of Avik [145], Rasoulinezhad et al. [146], Ogungbenle and Rufus [147], and Adeleye et al. [148], who have also discovered that environmental degradation has positive effects on *MR*. Furthermore, Uzar [149] offers specific support to our findings on the significant moderating role of *InQ* in reducing the impact of environmental degradation on the subject. However, *UR* is substantive to reduce *MR* by 0.302 per 1,000 infants; inflation instability is found to neutralize the impact of *UR* on *MR*. This might be due to the reduction of purchase power and increase in general prices that slow down the process of *UR*, affecting the *MR*. Finally, we found that, in addition to their moderating roles, both *InF* and *InQ* have direct effects on *MR*. While *InF* increases *MR*, *InQ* has a significant effect on reducing *MR* in South Asia.

### Robustness checks

The results obtained from the 2Sys-GMM model, as reported in Tables 3A–4B, demonstrate robustness. Each table includes diagnostic checks to ensure the reliability of the estimations. In particular, the results of Arellano-Bond (1) are significant at the 1% level, leading to rejection of the null hypothesis of no first-order autocorrelation. Conversely, the statistical results for Arellano-Bond (2) are insignificant to reject the null hypothesis of no second-order autocorrelation across all estimated models [150]. Additionally, we conducted tests for examining the overidentifying constraints through the Sargan-Hansen model. The results indicate that the combined null hypothesis of instrumental validity—meaning that the instruments are uncorrelated with the error term—and the appropriateness of excluding omitted instruments from the estimated equations holds [151].

## Conclusion

Economic disparities persist in our world, where stark contrasts exist between those succumbing to extreme hunger and deprivation of basic human rights and others facing health risks due to overeating. South Asia, as the second-poorest region globally, grapples with high rates of poverty, hunger-related deaths, and limited access to essential healthcare facilities. This study seeks to explore the health consequences of FI across eight South Asian countries: Afghanistan, Bangladesh, Bhutan, India, the Maldives, Nepal, Sri Lanka, and Pakistan. Spanning the years from 2000 to 2021, the study employs datasets compiled from various reliable sources. Notably, the study introduces innovative variables, including an inflationary shock variable (*InF*) and a composite institutional quality index (*InQ*), using the generalized autoregressive conditional heteroskedasticity and the distance-based approach index construction of Sarma [102], respectively. *InF* aims to capture the impact of global inflationary shocks on health outcomes, while *InQ* aims to assess how effective institutions can counteract and control *InF* alongside other local factors. To analyze the data, the study employs the 2Sys-GMM model as the primary functional equation.

The study’s results unveil compelling insights into the dynamics of life expectancy and infant mortality rates in South Asia. In particular, the prevalence of undernourishment (*PN*), inequality in per capita calorie intake (*CI*), CO_2_e, and *InF* emerge as influential factors with negative impacts on life expectancy but positive effects on infant mortality rates. These variables are found to play a significant role in reducing life expectancy and increasing infant mortality rates in the region. Conversely, per capita kilocalorie supply (*KS*), per capita GDP growth (*PG*), per capita health expenditures (*HE*), and urbanization (*UR*) are identified as significantly effective contributors to increasing life expectancy and decreasing infant mortality rates. The findings emphasize the negative moderating role of *InF*, accentuating the adverse effects of *PN*, *CI*, and CO_2_e on life expectancy, while amplifying their positive impacts on infant mortality rates. This underscores the direct and severe consequences of inflation instability on FI and environmental factors, leading to higher infant mortality rates and diminished contemporary life expectancy in South Asia. In rebuttal, the study employs *InQ* to examine its direct unconditional and moderating effects. *InQ* is revealed to have both positive and negative impacts on life expectancy and infant mortality rates, respectively. Moreover, *InQ* emerges as a significant moderator, effectively tempering the positive effects of *KS*, *PG*, *HE*, and *UR* on life expectancy, while mitigating the negative effects of *PN*, *CI*, and CO_2_e on the subject. The role of *InQ* extends to moderating the relationships between the variables and infant mortality rates, further highlighting its crucial influence in shaping health outcomes in South Asia.

### Policy implications

From the findings, two policy implications emerge as follows:

#### Triple constraints

The results identify three critical constraints—FI, environmental factors, and economic vulnerability to global shocks—that significantly impact both life expectancy and infant mortality rates in South Asia. Major FI constraints arise from insufficient food products to meet daily requirements. Given the region’s heavy reliance on agriculture, governments need to support farmers. Implementing specific financial and non-financial incentive schemes is essential to enhance contemporary agricultural capacity. This proactive approach is imperative in effectively combating extreme hunger and improving overall food security in South Asia. Environmental degradation poses alarming threats to life expectancy and infant mortality rates. Contributing factors include individual poverty, low-quality production machinery leading to industrial pollution, and inadequate governmental support at both public and private sector levels. A comprehensive strategy addressing these factors concurrently is necessary to improve environmental quality. Governments must focus on mitigating individual and industrial impacts, alongside providing robust support to environmental initiatives on a national scale. Inflationary shocks emerge as a significant factor affecting both life expectancy and mortality rates. While eliminating global shocks is challenging, the region can explore strategies such as fostering free trade and establishing visa-free zones. Promoting higher integration, increased trade, and enhanced capital exchange can help mitigate the adverse effects of inflationary shocks on health outcomes. By opening new avenues for cooperation and economic resilience, South Asian nations can better navigate and counteract the impacts of global economic fluctuations on public health.

#### Institutional constraint

The findings indicate that poor institutional quality hampers health outcomes, while its improvement proves effective in enhancing both the efficiency and scope of health outcomes. Significantly, institutional quality serves as a remedy to mitigate the negative effects of external shocks on contemporary endogenous FI and environmental indicators. The governments of South Asia urgently need to prioritize the promotion, advancement, and institutionalization quality factors within public organizations. This necessitates a focused effort to combat corruption, improve government efficacy, and advance other elements of good governance.

### Study’s limitations

The present study examined the effects of food insecurity and environmental degradation on life expectancy and mortality rates in South Asia, pinpointing specific policy areas that demand attention. However, two major limitations are acknowledged throughout writing this piece of investigation: firstly, the unavailability of health-sector-specific disaggregated datasets. Aggregate data provides a general overview of the current state; however, if disaggregated datasets were available, cross-sector specific results could help deeper insights into existing policies measures. Future studies, equipped with such datasets, may address these empirical shortcomings. Secondly, the overspecification issue. Due to this constraint, the present study could not incorporate additional explanatory variables such as health diplomacy, foreign direct investment, and domestic credit to private health sector, which could influence health outcome indicators. Subsequent studies could benefit from broader observations, integrating these predictors into their analysis for a more comprehensive understanding of the complex dynamics influencing health outcomes in South Asia.

## Data Availability

Datasets relevant to LE, MR, SE, PG, HE, and UR have been compiled from the World Development Indicators (WDI). Datasets for PN, KS, and CI were collected from UN-FAO. The data for CO_2_e has been collected from the Global Carbon Budget (2022). Finally, datasets for constructing InQ have been compiled from Worldwide Governance Indicators (WGI) sources.
